# Disorder-induced suppression of the zero-bias conductance peak splitting in topological superconducting nanowires

**DOI:** 10.3762/bjnano.9.128

**Published:** 2018-05-04

**Authors:** Jun-Tong Ren, Hai-Feng Lü, Sha-Sha Ke, Yong Guo, Huai-Wu Zhang

**Affiliations:** 1 State Key Laboratory of Electronic Thin Films and Integrated Devices and School of Physical Electronics, University of Electronic Science and Technology of China, Chengdu 610054, China; 2 Department of Physics and State Key Laboratory of Low-Dimensional Quantum Physics, Tsinghua University, Beijing 100084, China

**Keywords:** conductance peak spacing, disorder, Majorana energy splitting, shot noise, zero-bias conductance

## Abstract

We investigate the effect of three types of intrinsic disorder, including that in pairing energy, chemical potential, and hopping amplitude, on the transport properties through the superconducting nanowires with Majorana bound states (MBSs). The conductance and the noise Fano factor are calculated based on a tight-binding model by adopting a non-equilibrium Green’s function method. It is found that the disorder can effectively lead to a reduction in the conductance peak spacings and significantly suppress the peak height. Remarkably, for a longer nanowire, the zero-bias peak could be reproduced by weak disorder for a finite Majorana energy splitting. It is interesting that the shot noise provides a signature to discriminate whether the zero-bias peak is induced by Majorana zero mode or disorder. For Majorana zero mode, the noise Fano factor approaches zero in the low bias voltage limit due to the resonant Andreev tunneling. However, the Fano factor is finite in the case of a disorder-induced zero-bias peak.

## Introduction

Searching for Majorana bound states (MBSs) have recently received widespread attention due to their potential applications in topologically-protected quantum computing [1–9]. In the past two decades, the realizations of MBSs has been predicted in many condensed-matter systems, including *p*-wave superconductors [10–11], topological insulator-superconductor hybrid structures [12–13], artificially engineered Kitaev chains [14–15], semiconductor-superconductor hybrid nanowire systems [16–21]. Very recently, the one-dimensional Majorana mode running along the sample edge was shown in the heterostructure consisted of a quantum anomalous Hall insulator bar contacted by a superconductor [22]. Among all these proposals, the semiconductor-superconductor hybrid Majorana systems have attracted particular attention and have been demonstrated in several experiments since 2012 [23–30]. As an important signature of MBSs in the semiconductor nanowires which are proximity-coupled to *s*-wave superconductors, the zero-bias conductance peak has been observed in the tunneling spectra in the presence of a finite magnetic field [23–28]. However, it is suggested that such zero-bias features could also be induced by non-topological physics such as Kondo effect [31], smooth confinement [32], or strong disorder [33–35].

In one-dimensional case, the hybridization of the pair of MBSs localized at the wire ends produces a finite Majorana energy splitting and zero-bias peak splitting [36–38] due to the finite size effects. In a recent experiment [39], the energy splitting of Majorana zero mode has been observed in InAs nanowire segments with epitaxial aluminium, which forms a proximity-induced superconducting Coulomb island. It is illustrated that the energy splitting is exponentially suppressed with increasing wire length. For short wires with a typical length of a few hundred nanometers, the Majorana energies oscillate as the magnetic field varies. These observations are consistent with previous theoretical predictions [36–37]. However, there still exist some critical discrepancies between the theories and experimental results of the evidences for the MBSs. Firstly, it is easy to note that the zero-bias peak is significantly lower than the predicated value of 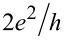, whereas the MBSs are expected to give exactly 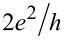 [40–43]. Secondly, theory predicts an increasing oscillation magnitude of Majorana energy splitting with the increase of magnetic field [36,44], while the experiment indicates the damped oscillation with increasing field. Similar discrepancy was also shown in the Majorana-quantum dot hybrid devices in the subsequent experiments [45–47]. It is important to know what physical mechanism leads to the damped oscillation of Majorana energy splitting.

Up to now, several theoretical studies have been devoted to explain these discrepancies [48–61], among which some possible reasons have been proposed, such as the combining effect of high temperature and multisubband occupancy in a Coulomb-blocked nanowire where the non-topological low-energy Andreev bound states and MBSs simultaneously exist [53], the zero-energy pinning effect induced by the interactions between the bound charges in the dielectric surroundings and the free charges in the nanowire [55], a finite leakage out of the Majorana modes due to the presence the normal drain [59], a finite coherence length in the induced superconducting pairing [60], and the orbital magnetic effects [61]. Although it is noticed that the trivial Andreev bound states are non-negligible in the experiments, the enhanced Majorana energy oscillation for increasing Zeeman field is robust and unaffected when various mechanisms are taken into account.

Here we investigate the effect of different types of disorder on the transport properties of a topological superconducting wire hosting a pair of MBSs. Although the disorder-modulated phase transition in this system has been widely discussed [43,62–74], we focus on the transport properties, especially the splitting of zero-bias conductance peak in presence of disorder. We adopt the non-equilibrium Green’s function (NEGF) method for a tight-binding model of the nanowire. Three different types of disorder are separately considered, including the disorder in the site-dependent chemical potential, the spatial deformations of the superconducting gap, and hopping disorder between the nearest neighbors. The results reveal that the disorder could significantly suppress the conductance magnitude. More importantly, the splitting of the conductance peak is removed by the disorder and a zero-bias peak is reformed with an increasing disorder strength. This paper is organized as follows. In section ’The model’ we present a tight-binding model for the one-dimensional superconducting nanowire and the theoretical framework based on NEGF. In section ’Numerical results’ we give the numerical results of the conductance and the noise Fano factor for different wire lengths and discuss different types of disorder-induced effect on these transport properties respectively. Finally, we conclude our results in section ’Conclusion’.

## Results and Discussion

### The model

**Figure 1:**
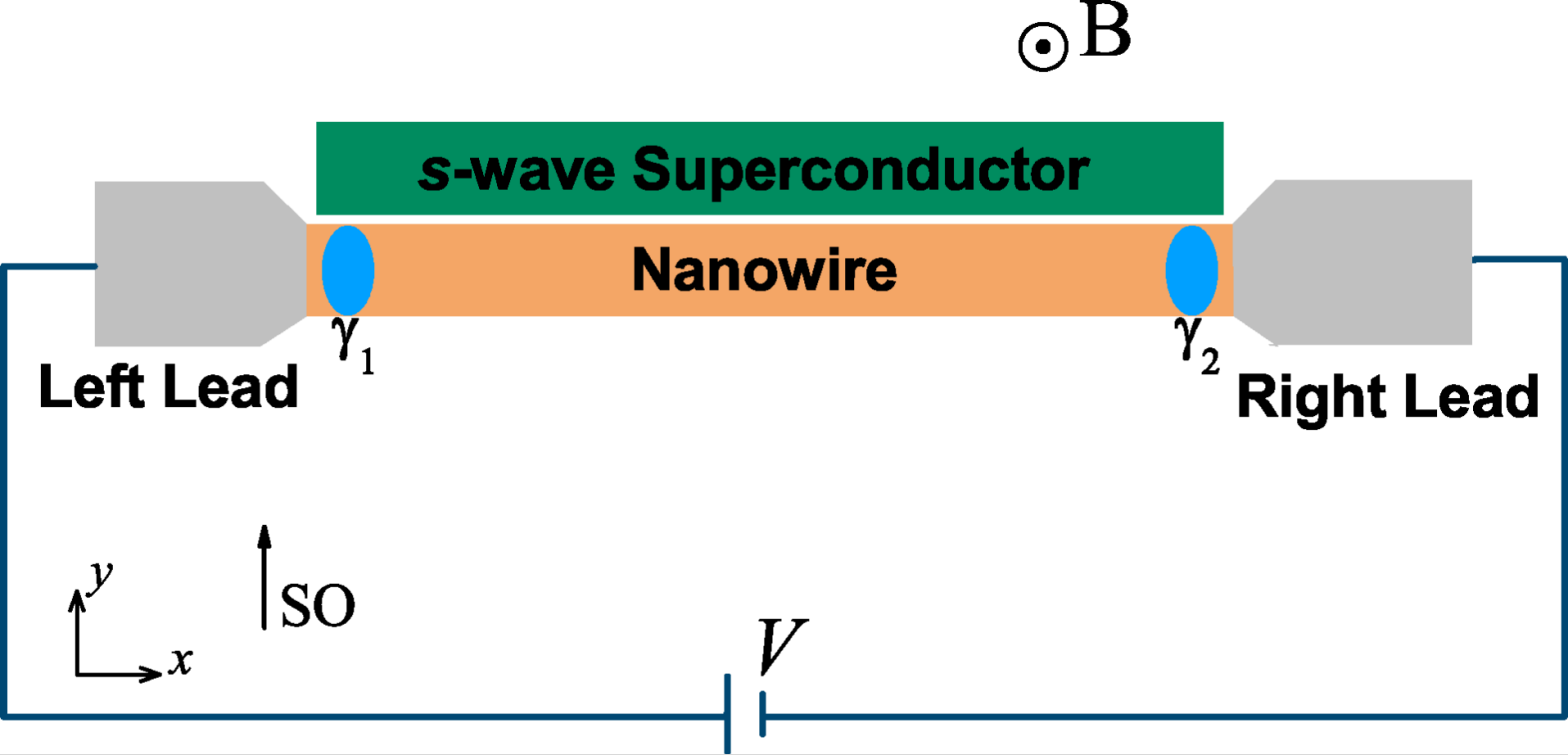
Scheme of our one-dimensional Majorana system. A semiconductor nanowire with spin-orbit interaction sandwiched by two normal leads (L, R) is proximity-coupled to an *s*-wave superconductor. The nanowire is driven into the topological phase and a pair of MBSs (γ_1_, γ_2_*)* emerge at the two wire ends with suitable parameters. A bias voltage *V* is applied across the device. The nanowire is arranged along the *x*-axis and the magnetic field (*B*) is applied along the *z*-axis, perpendicular to the spin-orbit coupling field (SO) in the *y*-direction.

The schematic representation of our one-dimensional Majorana system is shown in Figure 1. We consider a setup of two normal metal leads sandwiching a spin-orbit coupled semiconductor nanowire, which is covered by a parent *s*-wave superconductor to induce the proximity effect. The Zeeman field is realized by applying a magnetic field perpendicular to the spin-orbit coupling direction and the wire. It is proposed that such a hybrid system can hold a pair of MBSs at the two wire ends by tuning the Zeeman field or chemical potential to satisfy 
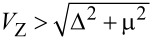
 [16–21], for which the nanowire will be driven into the topological phase. Here *V*_Z_, Δ and μ are the Zeeman splitting energy, proximity-induced superconducting pairing and the chemical potential, respectively. Although this work is motivated by the experiment by Albrecht et al. [39], our model does not take the Coulomb blockade effects into account. The reason is that the physics of disorder-induced suppression of the conductance peak spacings and reformation of the zero-bias peak, which we discuss below, is independent of Coulomb blockade physics. In the presence of a charging energy in the nanowire, it was shown that the zero-bias conductance values are considerably suppressed by the Coulomb energy [75]. The situation of interest to us is how intrinsic disorder in the nanowire affect the Majorana energy *E*_M_ and the splitted zero-bias conductance peak induced by *E*_M_. In situations like this, the intrawire charging energy could modulate the actual conductance value, but the main physics induced by the disorder is captured even though the charging energy is not taken into account.

The generic form of the Hamiltonian that models this Majorana hybrid structure reads as

[1]
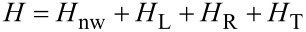


where the term *H*_nw_, *H*_L(R)_, and *H*_T_ account for the superconducting nanowire, the left (right) normal metal lead, and the tunnel coupling between the leads and the wire, respectively. Following the Bogoliubov–de Gennes formalism the Hamiltonian describing the low-energy physics for our one-dimensional superconducting wire is given by

[2]
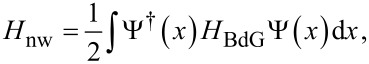


where 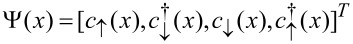 is the Nambu spinor for which *c*_σ_(x) 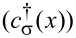 annihilates (creates) electrons with spin σ at position *x*. For numerical calculations, we invoke a lattice tight-binding model to discretize the BdG Hamiltonian and the Hamiltonian for the nanowire can then be written as [16–19]

[3]
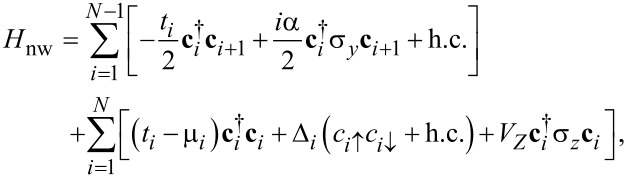


where *t_i_* characterizes the nearest-neighbor hopping between site *i* and *i* + 1, μ*_i_* and Δ*_i_* represent the on-site chemical potential and pairing, α is the spin-orbit coupling constant, **c***_i_* = [*c_i↑_*, *c_i↓_*]*^T^* (
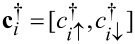
) is the spinor form of electron annihilation (creation) operator on the *i*th site, and σ*_i_*, *i* = 0, *x*, *y*, *z*, are Pauli matrices acting on the spin space. The wire length is *L* = *Na* where *a* is the lattice constant and *N* is the total number of sites. In this work, three different types of intrinsic disorder in the nanowire are considered: the fluctuations of the site-dependent chemical potential, the nonlinear tunneling between neighboring sites, and the disorder arising in the pairing as a result of inhomogeneous superconductor–semiconductor coupling. In the case of a clean wire, we set μ*_i_* = μ_0_, Δ*_i_* = Δ_0_, and *t_i_* = *t*_0_ for all sites. For each single disordered configuration of the system, the on-site disorder are modeled by the white noise and their strength is assumed to be randomly distributed in the range [−δ*W*, +δ*W*], where the *W* = *t*, μ, Δ denotes the strength for different types of disorder.

The Hamiltonian describing the normal metallic leads is given by

[4]
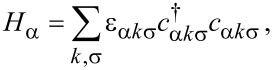


where ε_αkσ_ (α = L, R) represents the single-particle energy in the lead α and *c*_αkσ_ (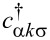) is the annihilation (creation) operator for the lead α. The sum is over momentum *k* and the spin σ. The last term in the total Hamiltonian, *H*_T_, characterizes the coupling between the wire and the two leads, which is given by

[5]
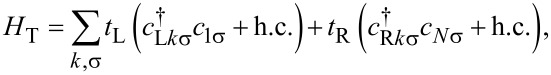


where *t*_L(R)_ denotes the hopping strength through left (right) lead and the wire. The operators *c*_1σ_ and *c_N_*_σ_ correspond to the annihilation operators on the first and last site at opposite ends of the wire. Taking all lattice sites into account, we can now write out the Hamiltonian for the nanowire as a 4*N* × 4*N* matrix of which the submatrix entry *H_i_*_,_*_j_* fully characterizes the coupling between site *i* and site *j*. The nonzero off-diagonal entries read as

[6]
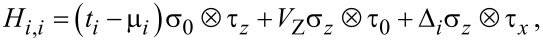


[7]
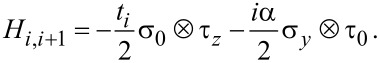


and the subdiagonals are related to the superdiagonals by 
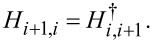
 Here τ*_i_*, *i* = 0, *x*,*y*,*z,* are the Pauli matrices acting on the Nambu space.

The operator of tunneling current from the lead α to the central region is defined as


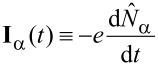


and then one can obtain [76–79]

[8]
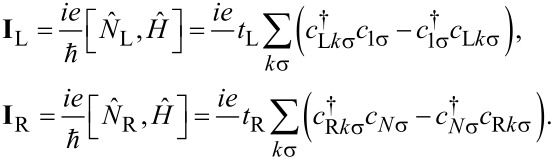


The current noise correlations are defined as

[9]
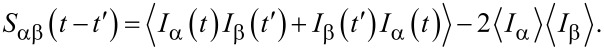


*S*_αβ_ is referred to as the noise auto- or cross-correlation between the currents flowing through the lead α and lead β. To evaluate the current and noise within the framework of Keldysh NEGF formalism, we need to derive the retarded (advanced) Green’s function *G^r^*^(^*^a^*^)^ and the lesser (greater) Green’s function *G^<^*^(^*^>^*^)^ from the contour-ordered Green function 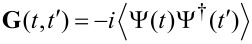 in the Nambu space spanned by the spinor 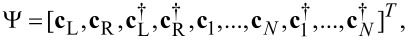 where **c**_L(R)_ is the electron annihilation operator in the left (right) lead. In this Nambu space, we define the matrix of the lesser Green’s function **G***^<^* [78–79]

[10]
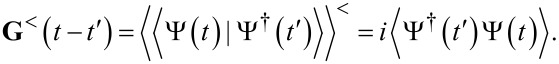


In this representation, the currents are given by

[11]
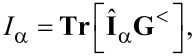


and the noise spectrum *S*_αβ_(ω) is given by:

[12]
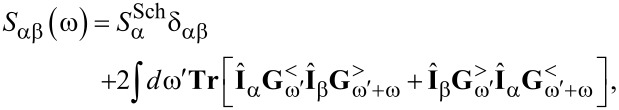


where 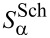 is the frequency-independent Schottky noise originating from the self-correlation of a given tunneling event with itself, which the double-time correlation function can not contain, and 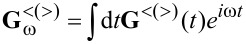 denotes the lesser (greater) green functions in the frequency space. The matrices of the current operators are given by

[13]
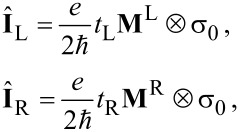


where **M**^L^ and **M**^R^ are the block (2*N* + 4) × (2*N* + 4) matrices with nonzero elements

[14]
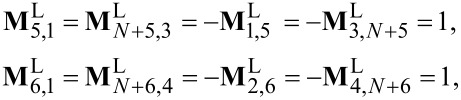


respectively. From the standard equation of motion for the central region, we can write the retarded Green’s function **G**^r^ in terms of the Dyson equation **G**^r^ = **g**^r^ + **g**^r^Σ^r^**G**^r^, which gives

[15]
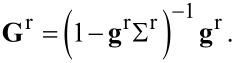


Here **g**^r^ is the bare Green’s function of the central region without coupling to the leads (*t*_L_ = *t*_R_ = 0),

[16]
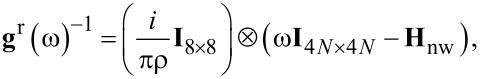


where **I***_n_*_×_*_n_* is the *n* × *n* identity matrix. Since **G**^r^ is already given and the advanced Green’s function **G**^a^ can be obtained from **G**^r^ = (**G**^a^)^†^, it is now straightforward to obtain the lesser Green’s function from the standard Keldysh equation,

[17]
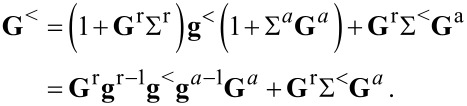


In the present case, Σ*^<^* = 0 and

[18]
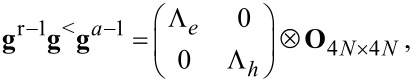


with

[19]
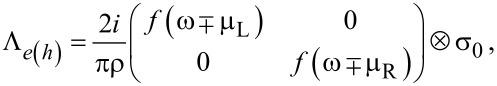


where **O**_4_*_N_*_×4_*_N_* is the 4*N* × 4*N* zero matrix, 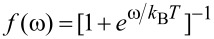 is the Fermi–Dirac distribution function and *k*_B_*T* is the temperature. In the calculation of the noise spectrum *S*_αβ_(ω), the greater Green’s function **G***^>^* can be readily obtained since the relation **G***^<^* −**G***^>^* = **G**^a^ −**G**^r^ holds. Finally, we define the noise Fano factor *F* = *S*_L_(ω = 0)/2*eI*_L_ to measure the deviation from the uncorrelated Poissonian noise for which *F* = 1, with respect to which the shot noise can be enhanced or suppressed because the current fluctuations in the device are highly susceptible to different interactions in the system.

### Numerical results

In this section we present the numerical results of the transport properties for the disordered Majorana nanowire. Here we mainly discuss the disorder-induced effects on the differential conductance, especially on the conductance peak spacing and its relation with the Majorana energy oscillation. To exclude thermal fluctuations, we restrict our discussion to the zero temperature *k*_B_*T* = 0. The lattice constant is set to *a* = 10 nm throughout the paper. For the disorder-free situation, we choose *t*_0_ = 12 meV, μ_0_ = 2.0 meV, Δ_0_ = 0.9 meV, α = 2.4 meV, and the symmetric lead-wire coupling strength Γ_L_ = Γ_R_ = 0.3 meV. The bias voltage *V* across the whole device will shift the chemical potential μ_L_(μ_R_) in the leads to ±*V/*2. In modeling the disorder effect on the quantum transport in mesoscopic devices, the numerical results need to be averaged over enough random configurations. In our calculation, the conductance and the noise Fano factor is averaged over 400 random configurations for each data point.

In previous work [35], it was found that the disorder could induce a nonquantized zero-bias peak at finite temperature even when the nanowire is in a topologically trivial regime. In their work, a single disorder realization is considered for their 3-dimensional multiband Majorana wire. The consideration of the multiband wire model leads to the weaker sample–sample fluctuations than the single channel model. Although a single disorder configuration is considered, their results are obtained at a finite temperature, which implies that thermal averaging is done. With the increase of temperature, the sample-to-sample fluctuations are suppressed [80]. It is thus reasonable for them to consider a single disorder configuration.

Here we study the effect of three types of disorder on the transport in a Majorana device. To exclude the thermal effect, we restrict our discussion to the zero temperature case. The large sample-to-sample fluctuations is thus unavoidable. In principle, several similar samples are also needed in experiments to confirm the existence of related physical mechanisms. In a previous experiment [39], only one sample is reported for each wire length. It is indicated that a damped oscillation magnitude of the Majorana energy splitting occurs with the increase of magnetic field, which contradicts the theoretical result. Our calculation suggests that the discrepancy may arise from the intrinsic disorder. To confirm this, more experiments are expected to be performed in the future for similar samples.

#### Majorana energy oscillation

**Figure 2:**
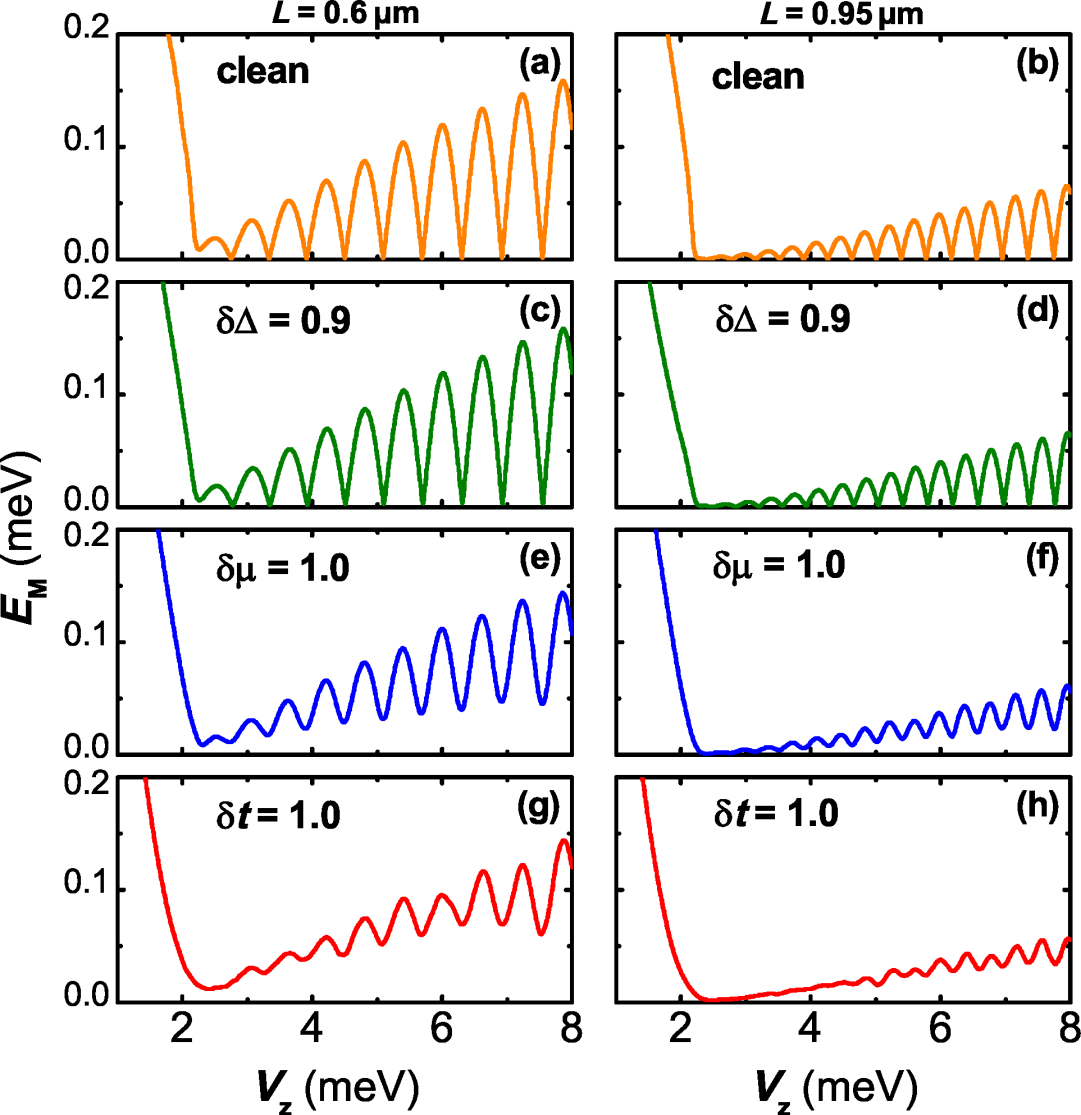
The Majorana energy *E*_M_ as a function of the Zeeman splitting *V*_Z_ for different types of disorder. (a,b) The clean cases; (c,d) disorder in pairing energy δΔ = 0.9 meV; (e,f) disorder in the chemical potential δμ = 1.0 meV; (g,h) disorder in the nearest hopping δ*t* = 1.0 meV. For comparison, two different wire lengths *L* = 0.6 μm (left panels) and *L* = 0.95 μm (right panels) are separately considered. Other parameters are taken as *t*_0_ = 12.0 meV, Δ_0_ = 0.9 meV, μ_0_ = 2.0 meV, α = 2.4 meV, and Γ_L_ = Γ_R_ = 0.3 meV. The MBSs appears at the wire ends for *V*_Z_ *> V*_ZC_.

We firstly present the lowest energy *E*_M_ as a function of the magnetic field in the presence of different kinds of disorder. Considering the finite-size effects on the coupling between the two MBSs and the recently reported suppression of the energy splitting due to the increase in wire length [39], we consider wires of two typical lengths in particular: a shorter one with *L* = 0.60 μm and a longer one with *L* = 0.95 μm. In Figure 2, when *V*_Z_ is relatively small, the system stays in the topologically trivial phase, and the lowest energy is linearly suppressed as the magnetic field strength increases. Without disorder in the system, the nanowire is driven into a topological superconducting phase when we tune *V*_Z_ to exceed the phase transition point 
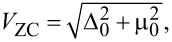
 and *E*_M_ begins to oscillate near the zero value. This behavior, originating from the finite-size effects, is absent in a long enough wire, where the field-independent exact Majorana zero mode emerges with its energy pinned to zero.

For disordered wires, we find that the exact Majorana zero mode gradually vanishes in the presence of disorder in hopping or chemical potential. In particular, as shown in Figure 2, a δ*t* with strength 1.0 meV, which is comparable to the strength of Zeeman splitting, can remarkably flatten the energy oscillation. On the contrary, the strong disorder in the pairing energy leaves the Majorana energy oscillation almost unaffected. In the topological phase and in the strong Zeeman field regime, the spins are nearly polarized and one can project the original Hamiltonian onto a simpler one-band problem [7]. To leading order, one obtains an effective *p*-wave-like Hamiltonian with the effective chemical potential μ_eff_ = μ + *V*_Z_/2 and the effective pairing energy Δ_eff_ = αΔ/2*V*_Z_. Because small spin-orbit coupling is considered, the effect of the disorder δΔ in the pairing energy is considerably suppressed with increasing *V*_Z_ due to the multiplication factor α/2*V*_Z_. However, there is no multiplication factor for μ, hence the disorder δμ has a stronger influence on the Majorana energy oscillation. The hopping disorder and chemical potential disorder can both considerably destroy the Majorana zero modes, leading to increased Majorana energy splitting and enhancement of the MBSs hybridization.

To get a closer look into the effects of disorder on the Majorana energy splitting, it is beneficial to investigate the localization length that characterizes the hybridization between the pair of MBSs. In weak spin-orbit coupling regime, the localization length increases gradually as 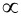
*B* [36,38]. Therefore, the strength of the Zeeman splitting *V*_Z_ should be chosen as the energy scale to determine whether the disorder strength is strong or not. Meanwhile, the disorder strength that can remove the energy splitting signature is also determined by the wire length. For a longer wire, a disorder of the same strength could lead to a more evident suppression of the energy splitting signature.

**Figure 3:**
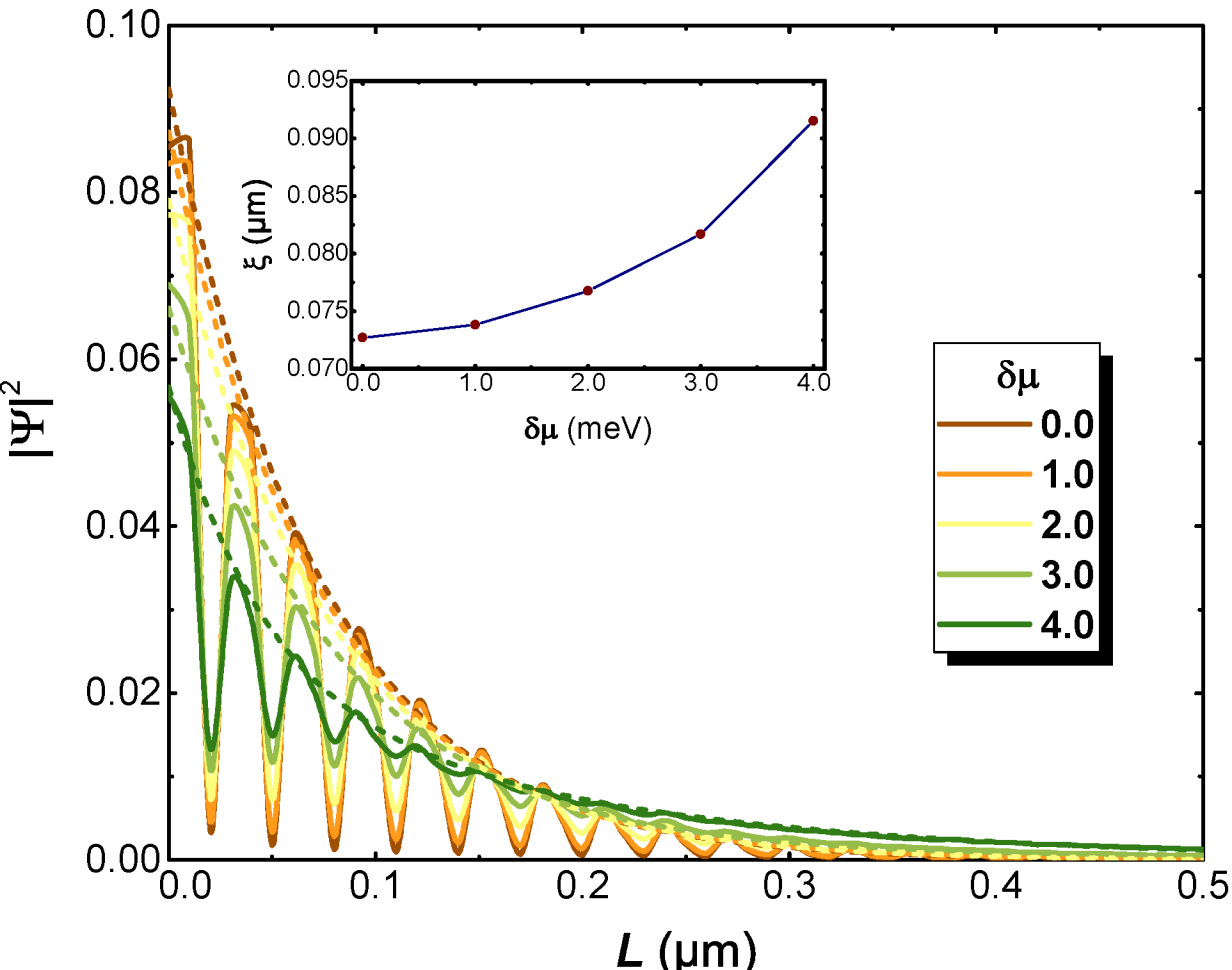
The spatial distribution of probability density |Ψ|^2^ (solid lines) and their fitted envelopes 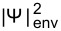 (dashed lines) in the presence of different strengths of chemical potential disorder δμ; the inset shows the localization length ξ of the fitted envelope varies with different values of δμ. Here we choose *L* = 2.0 μm, μ = 0, Δ = 2.0 meV, *V*_Z_ = 6.1 meV and other parameters are taken as those used in Figure 2.

In Figure 3, without loss of generality, we focus on the evolution of the MBS probability density on the left wire end in the presence of disorder in chemical potential, of which the influence is more evident compared with the limited effects induced by the pairing disorder. Here we choose a rather long wire of length *L* = 2.0 μm, where the two spatially separated MBSs are well localized at each end of the wire, thus the hybridization between the pair of MBSs is negligibly small. In our case where 
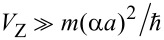
 the system is in a weak spin-orbit interaction regime, and the approximate value of the localization length for a discretized tight-binding model is analytically given by


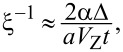


with which the MBS probability density has an exponentially decaying envelope of the form 
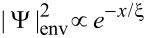
 [81]. As shown in Figure 3, the numerically fitted decaying envelope of the disorder-free probability density gives ξ ≈ 0.0727 μm, compared to the approximate analytical results of ξ ≈ 0.0775 μm the difference is below a lattice constant. With the disorder strength increasing, the probability density at the end is suppressed and the localization length ξ of the fitted envelope becomes larger. This can also be directly identified from the noticeable deformations of the tail part of the probability density, which implies an enhanced hybridization between the two MBSs with an increasing disorder strength. In a shorter wire where the overlap between the two MBSs is stronger, it is reasonable to expect a more evident disorder-induced increment in the MBSs hybridization, which agrees with the results shown in Figure 2.

#### Conductance peak spacings

**Figure 4:**
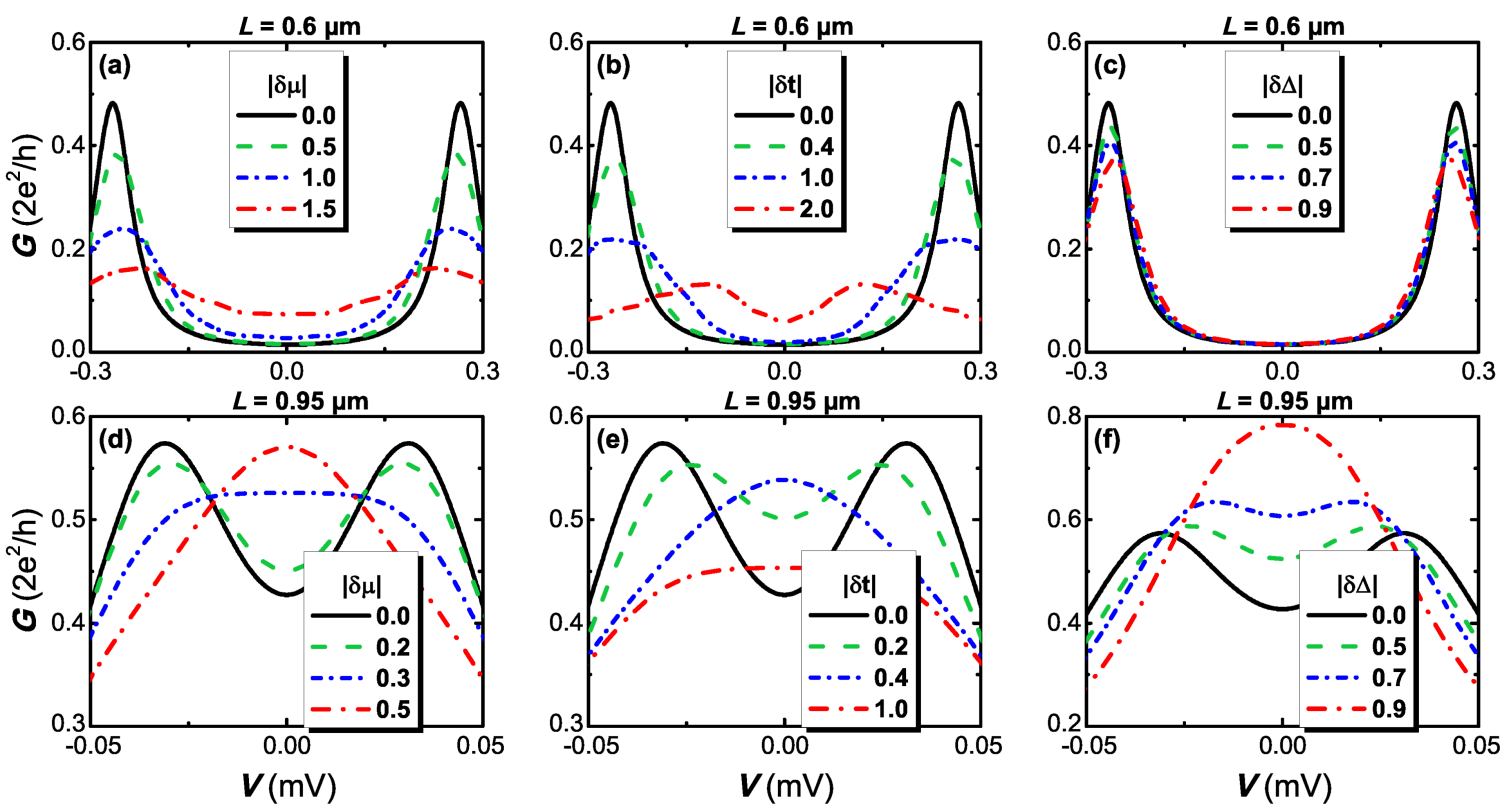
The differential conductance *G* = d*I*/d*V* as a function of the bias voltage *V* under the influence of different types of disorder. (a,d) disorder δμ in the chemical potential; (b,e) disorder δ*t* in the nearest hopping; (c,f) disorder δΔ in pairing energy. The upper panels corresponds to the shorter wire case *L* = 0.6 μm and the lower panels represents the case of *L* = 0.95 μm. Other parameters are taken as those used in Figure 2.

In Figure 4, we demonstrate the effects of three types of disorder on the conductance peak spacings for different wire lengths. In a disorder-free case, the Majorana energy splitting of the system can be reflected by the conductance peak spacing. We take a Zeeman field *V*_Z_ = 6.6 meV that is associated with clear energy splittings and conductance peak spacings.

For a shorter nanowire *L* = 0.6 μm, it is found that all three types of disorder can suppress the amplitude of the conductance peak and broaden the peak width to some different extent. The presence of disorder in the system leads to a similar result induced by dissipation or finite temperature, both of which can lower the peak and broaden its width [57]. What makes a difference here is that one can additionally observe a suppression, which is pronounced especially in the cases of hopping or chemical potential, of the conductance peak spacings due to the effect of disorder.

When the device becomes longer (*L* = 0.95 μm), the Majorana energy splitting is exponentially suppressed, thus the conductance peak spacing in a clean system becomes much narrower. As illustrated in the lower panels of Figure 4, smaller disorder than that in the shorter wire can lead to notable suppressions on the conductance peak spacings, and as the disorder strength eventually exceeds some certain value, a zero-bias peak is formed from the two spaced peaks. It is interesting that a strong disorder in pairing could even elevate the induced zero-bias conductance peak. These numerical results, together with that revealed in Figure 2, suggest that we can not simply neglect the role played by disorder in detecting Majorana energy oscillation experimentally through transport measurements since for some values of Zeeman field the disorder-induced effects can broaden the Majorana energy splitting of the low-energy states while simultaneously narrows the conductance peak spacing. This means that the Majorana energy splitting can not be genuinely characterized by the conductance signature. One possible reason is that the Majorana energy splitting is not robust. When the energy splitting of the Majorana modes is negligible compared to the magnitude of disorder, the conductance signature associated with the Majorana energy splitting could be annihilated by the noise arising in the system, which is equivalent to raising the temperature. Different from the thermal fluctuations that could be excluded by lowering the temperature, the three types of intrinsic disorder discussed here are hard to avoid in a realistic experiment.

**Figure 5:**
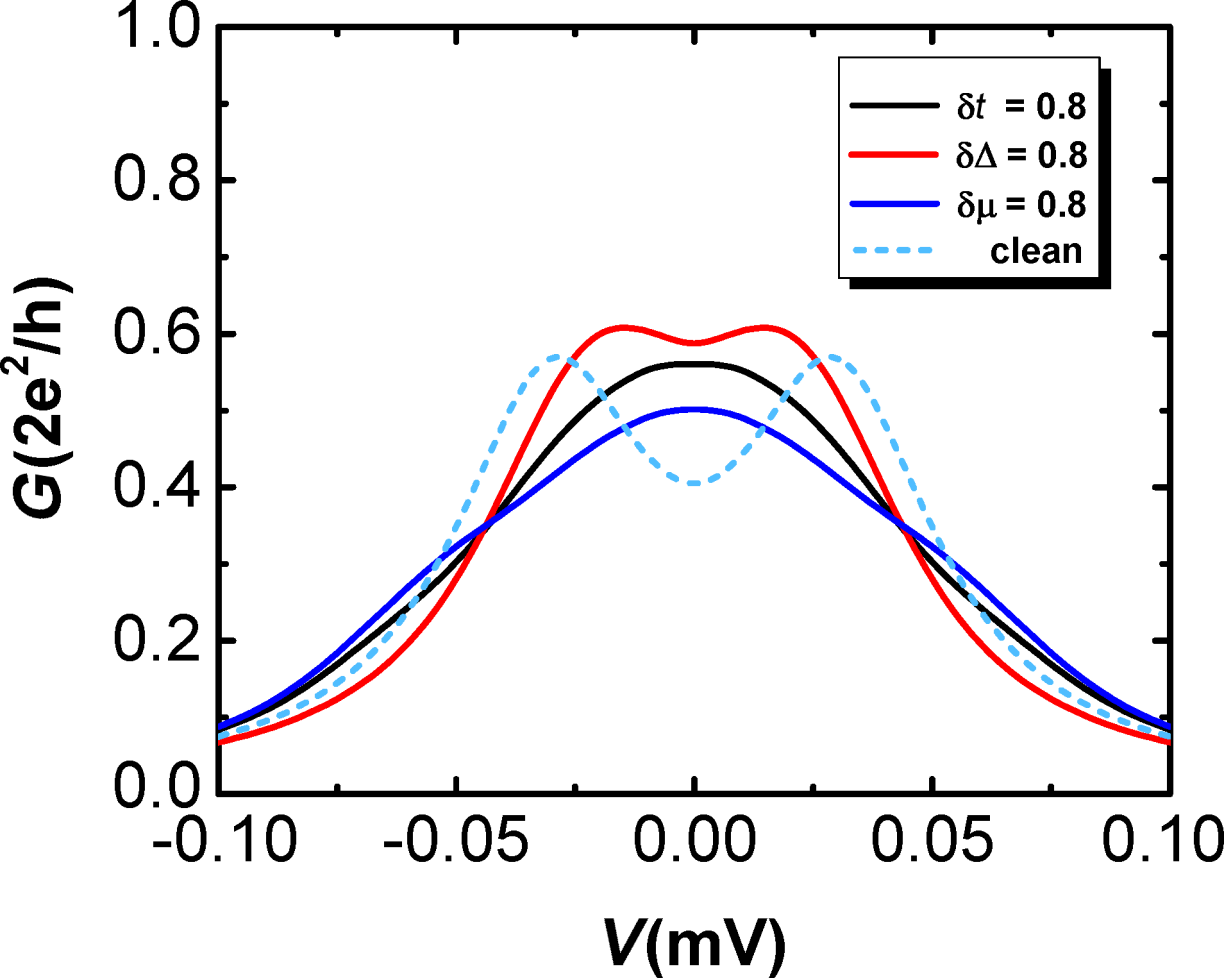
The differential conductance *G* = d*I*/d*V* in the longer wire (*L* = 0.95 μm) as a function of the bias voltage *V* with δμ = δΔ = δ*t* = 0.8 meV approaching the critical Zeeman splitting *V*_ZC_ = 0.9 meV. Here we have μ_0_ = 0, *V_Z_* = 6.0 meV and other parameters are taken as those used in Figure 4.

Above we consider the case that the critical Zeeman field *V*_ZC_ is much stronger than the disorder strength δ*W*. Now we turn to discuss the more experimentally relevant case where δ*W* ≈ *V*_ZC_. Figure 5 demonstrates the effect of three types of disorder on the conductance for a small *V*_ZC_. The chemical potential in the wire is tuned as μ_0_ = 0, while the other parameters are taken as the same as that for the lower panels in Figure 4. It is shown in Figure 5 that for δ*W* ≈ *V*_ZC_, the disorder can suppress the peak spacing and a single zero-bias peak is produced. Similar to the large *V*_ZC_ case, the main conclusion is qualitatively consistent with the results in Figure 4.

In previous experiments [39,46–47], the Majorana energy splitting for a nanowire with Coulomb interactions was represented by the even–odd peak spacing differences. However, the expected field-dependent decay behavior of Majorana energy oscillations is not observed in the experiments. On the contrary, the detected conductance peak differences tend to decay sharply as the magnetic field increases, which contradicts the theoretical predictions. Although here we consider an interaction-free scenario, our results indicate that the disorder can partially reduce the splitting of the conductance peak. In addition, for a shorter wire, the hybridization of the MBSs at two ends can generate a relatively large splitting in the conductance peak, which is consistent with the result of the previous experiments. The magnetic field could suppress the superconducting pairing energy, which leads to the enhancement of disorder strength in some sense.

#### Zero-bias conductance as a function of Zeeman field

**Figure 6:**
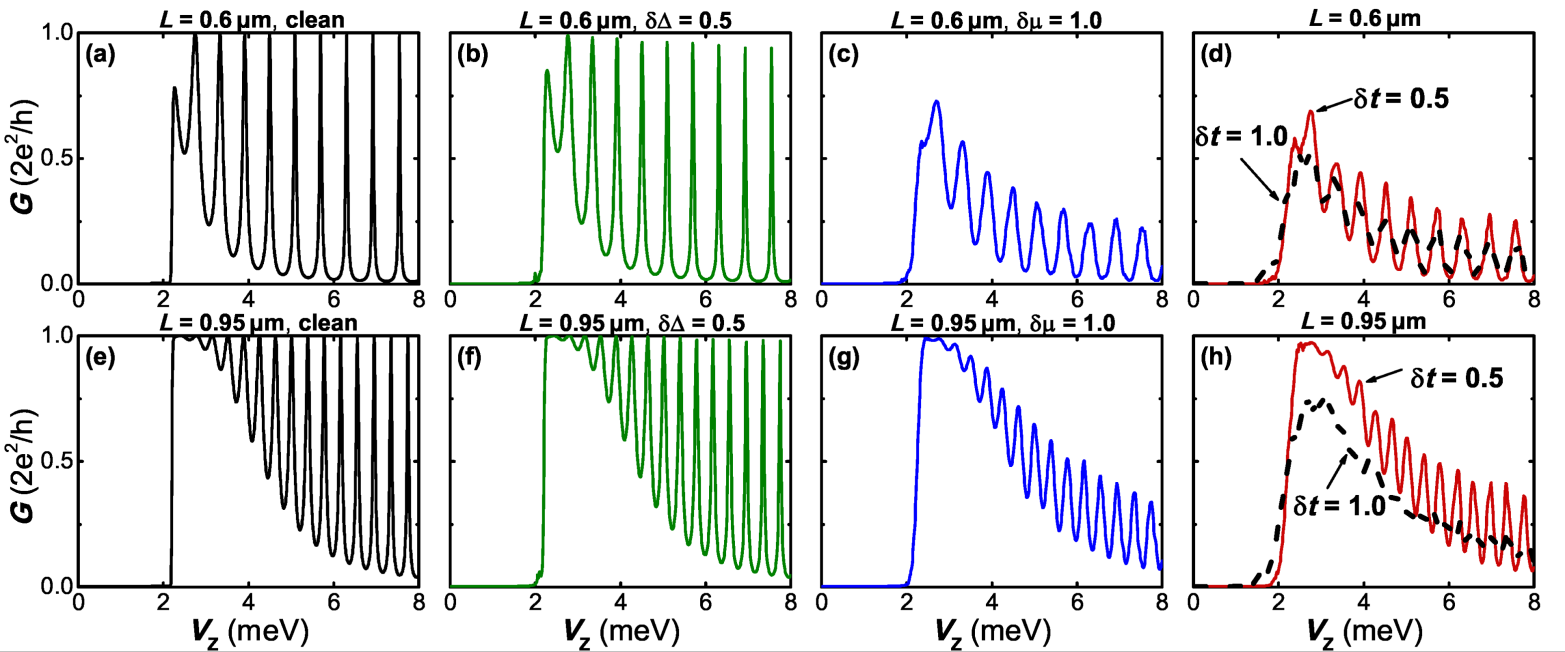
The zero-bias conductance *G* as a function of the Zeeman splitting *V*_Z_ for different types of disorder. (a,e) The clean case; (b,f)disorder in pairing energy δΔ = 0.5 meV; (c,g) disorder in the chemical potential δμ = 1.0 meV; (d,h) disorder in the nearest hopping δ*t* = 0.5 meV and 1.0 meV. The upper panels correspond to the shorter wire case *L* = 0.6 μm and the lower panels represent the case of *L* = 0.95 μm. In panel (h), we show that a disorder of δ*t* = 1.0 meV could remove the conductance oscillation as *V*_Z_ increases. Other parameters are taken as those used in Figure 2.

In superconducting nanowire systems, a quantized zero-bias conductance peak is considered as direct evidence for the presence of MBSs, and its emergence is often associated with the resonant Andreev reflection [41]. However, for realistic Majorana nanowires, the observed conductance peaks are often much smaller than 2*e*^2^/*h*. In Figure 6, we show the disorder-induced effects on the zero-bias conductance oscillation as a function of the Zeeman splitting *V*_Z_. For the clean wire, the zero-bias conductance has a clear oscillating behavior in the topological phase (*V*_Z_ *> V*_ZC_), and its peak value is quantized to 2*e*^2^/*h*. These quantized peaks of the conductance emerge from the exact zero-energy modes, while the valley of the conductance corresponds to the peak value of Majorana energy splitting. With an increasing magnetic field, the valley of the conductance gradually decays, corresponding to an enhancement of the Majorana energy splitting through the magnetic field. When the magnetic field is strong enough, the transport channel of the resonant Andreev reflection is almost closed and the valley of conductance approaches zero.

In the presence of disorder, the most notable difference is that the conductance oscillation peaks do not become more quantized. In Figure 2, it is shown that the disorder could destroy the exact Majorana zero mode and produce a finite energy splitting. Correspondingly, the quantized zero-bias conductance peak is suppressed by the disorder, as a manifestation of the induced finite energy splitting. This phenomenon is particularly evident for the cases where the disorder in the hopping or in chemical potential exists. As shown in Figure 6b and Figure 6f, the conductance peaks stay almost quantized even in the presence of a relatively strong pairing disorder. Additionally, one can find that the valleys of the conductance oscillation are almost unaffected by all kinds of disorder, which also agrees with the result of Figure 2. These observations suggest that the intrinsic disorder in the nanowire could strongly reduce the zero-bias conductance oscillation associated with the Majorana energy splitting. However, although the disorder significantly suppresses the oscillation, it does not eliminate the zero-bias conductance peak.

#### Shot noise

**Figure 7:**
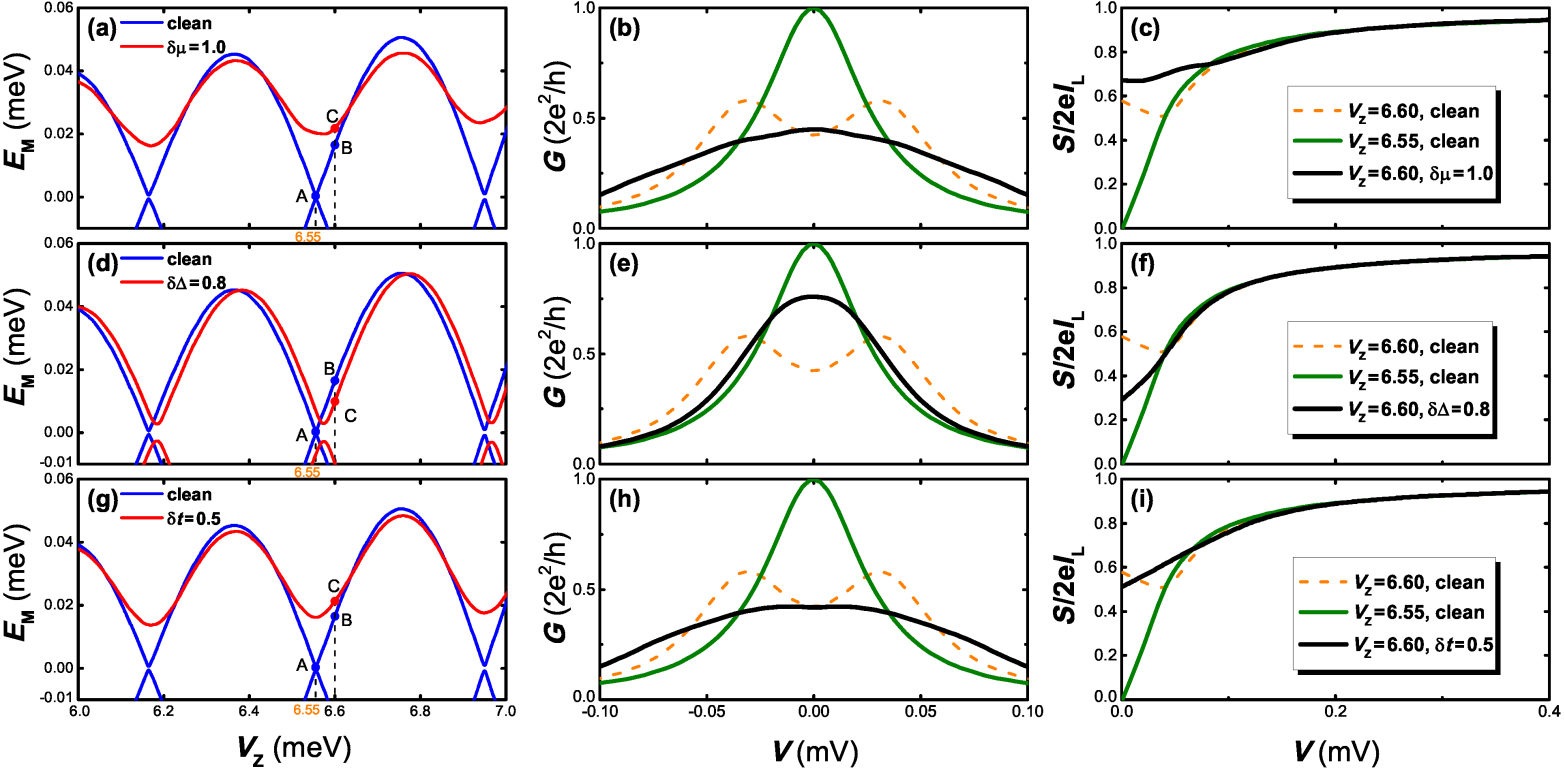
Comparison of the noise Fano factor *F* between the cases of Majorana zero mode and disorder-induced zero-bias conductance peaks. The upper, middle, and lower panels represent the effect of disorder in chemical potential δμ, superconducting pairing δΔ, and the hopping amplitude δ*t*, respectively. (a), (d) and (g): Majorana energy *E*_M_ as a function of *V*_Z_. The points A and B denote the MBSs with zero energy and a finite energy in the disorder-free case, respectively. The points C corresponds to the MBSs with a finite energy splitting in the disordered cases, where *V*_Z_ at point C equals to that at point B. (b), (e) and (h): The differential conductance *G* as a function of the bias voltage *V*. In the clean case, the quantized zero-bias peak is formed for Majorana zero mode (green lines). Disorder-induced zero-bias peaks (black lines) are formed from the spaced conductance peaks (orange, dotted line). (c), (f) and (i): The noise Fano factor *F* as a function of the bias voltage *V*. In the clean case, *F* in the low bias limit approaches zero for Majorana zero mode (green line), and *F* is finite for a finite energy splitting (orange, dotted line). For the disordered case, *F* in the low bias limit is finite (black line) although a zero-bias peak emerges in this case. The disorder strengths are δμ = 1.0 meV, δΔ = 0.8 meV, and δ*t* = 0.5 meV. The wire length is taken as *L* = 0.95 μm and other parameters are taken as those in Figure 2.

We now turn to investigate the shot noise properties of the Majorana system. For a long nanowire, the Majorana energy splitting is negligible, and the noise Fano factor is suppressed at zero voltage due to the resonant Andreev tunneling in an isolated MBS. In the clean case, a large Majorana energy splitting could strongly suppress the resonant Andreev tunneling, leading to the increase of the noise Fano factor and splitting of the conductance peak. It is shown in Figure 4 that the split conductance peaks are reformed to one zero-bias peak by the disorder. However, the zero-bias conductance peak can also arise due to the exact Majorana zero mode in the clean case. It is expected that the shot noise may provide the signature to distinguish the zero-bias conductance peak in a clean system from that which arises in a disordered one. This can be verified by the results given in Figure 7.

Here we present the Majorana energy splitting *E*_M_, the conductance *G* and the noise Fano factor *F* = *S/*2*eI* in the clean and disordered cases, in which three different types of disorder are taken into account. In the clean case, we separately choose point A and B which represents the zero energy mode and a finite splitting case, respectively. For a Majorana zero mode, a quantized zero-bias conductance peak could be induced and the noise Fano factor approaches zero due to the resonant Andreev tunneling. While for the case of finite energy splitting, the zero-bias conductance peak is split and the shot noise is enhanced due to the crossed Andreev reflection (CAR) which, contrasting with the local Andreev reflection that injects a Cooper pair in a single lead, would split a Cooper pair over two leads. The CAR processes will induce a current noise cross-correlation between two normal leads and predominate over the local Andreev reflection with the presence of a MBSs pair [77–79]. For short wires, the Fano factor at zero bias is close to unity for a strongly coupled MBS pair between two leads. As the wire length increases, the coupling between the MBSs at the two ends decreases, leading to the suppression of CAR process and a reduction of Fano factor.

For comparison we also choose a point C for the disordered case. The points C correspond to MBSs with a finite energy splitting in the disordered case, where *V*_Z_ at point C is equal to the Zeeman field at point B. As shown in Figure 7 the Majorana energy splitting in point C has a non-zero value, and its value is slightly enhanced or weakened with respect to the Zeeman field strength. For the conductance, the peak splitting at point B is reformed to a single zero-bias peak induced by three types of disorder. Differently, in the low-bias voltage regime, the noise Fano factor *F* deviates from zero in the presence of disorder, indicating a stronger coupling between the two separated MBS. This result is a clear manifestation of the Majorana-assisted CAR process. This means that although the zero-bias conductance peak could originate from an exact zero mode or intrinsic disorder in the nanowire, one can discriminate these two different mechanisms from the shot noise properties. In a clean nanowire, the zero-bias peak is induced by the Majorana zero mode. In this case, the appearance of the zero-bias peak is always accompanied by the zero noise Fano factor, i.e., *F* = 0. However, in the disordered case, the zero-bias conductance peak could also be induced for a finite energy splitting, while the noise Fano factor *F* has a finite value. Thus, whether the Fano factor *F* at the low-bias limit equals to zero or a finite value provides a signature to distinguish the zero-bias peak induced by Majorana zero mode from that by disorder.

## Conclusion

To conclude, we investigated the effect of intrinsic disorder on the transport properties of a Majorana nanowire by adopting a one-dimensional tight-binding model. We introduce three types of disorder into the system, including random fluctuations in the chemical potential, spatially changing in the superconducting pair potential, and the anisotropy of the nearest-neighbor hopping strength through lattice sites. We demonstrated that the disorder could remove the peak spacing in the differential conductance and induce a zero-bias peak for a finite Majorana energy splitting. For a shorter nanowire, the magnitude of the conductance peaks and the peak spacings are considerably suppressed as the disorder is taken into account. Such a disorder-induced suppression of conductance peaks and peak spacings provides a simple but interesting scenario to explain the absence of Majorana energy oscillation observed in previous experiments. Especially for a longer nanowire (*L* ≈ 1 μm), the Majorana energy splitting is exponentially small, and the spaced conductance peaks are facilitated to form a zero-bias peak by the disorder. However, the presence of disorder does not suppress the Majorana energy splitting. On the contrary, the disorder in hopping and chemical potential destroys the localization of MBSs and thus enhance their hybridization, leading to an increase in the Majorana energy splitting. This phenomenon can be further identified with the disorder-induced increment in the localization length. The exact Majorana zero modes in the clean case gradually vanish with increasing disorder strength. As a function of Zeeman field, the quantized zero-bias conductance peaks by the exact zero mode are shown to be strongly suppressed due to the presence of disorder. In particular, for an increase in hopping disorder, the oscillation behavior in the zero-bias conductance spectra vanishes in the longer wire case.

In the presence of disorder, the Majorana energy splitting is not suppressed and zero modes are removed, while the zero-bias conductance peaks are induced for a finite energy splitting. To distinguish whether the zero-bias conductance peak is induced by a Majorana zero mode or by the disorder, we further investigate the shot noise properties of the device. For a clean nanowire, we show that the appearance of the zero-bias peak is always accompanied by a zero-noise Fano factor (*F* = 0) in the low-bias voltage limit. In contrast, the Fano factor *F* in the disordered case has a finite value at the low-bias limit. In this case, the finite Majorana energy splitting induces a crossed Andreev reflection and the resonant Andreev tunneling is suppressed, resulting in the deviation of the Fano factor from zero. Therefore, the shot noise provides a clear signature to discriminate between the two different mechanisms that lead to the formation of the zero-bias conductance peak.
